# The Nuclear Transporter Transportin-3 Functions Under Oxidative Stress

**DOI:** 10.3390/cells15080708

**Published:** 2026-04-17

**Authors:** Megan A. L. Barling, David R. Thomas, David A. Jans, Kylie M. Wagstaff

**Affiliations:** Biomedicine Discovery Institute, Monash University, Clayton, VIC 3800, Australia

**Keywords:** transportin-3 (TNPO3), importin-13 (IMP13), nucleocytoplasmic transport, oxidative stress, confocal laser scanning microscopy (CLSM), Fluorescence Recovery After Photobleaching (FRAP)

## Abstract

Nuclear transport is a vital system that mediates movement of essential biomolecules between the nucleus and cytoplasm. It is tightly regulated by the Importin (IMP) superfamily to maintain separation of cellular compartments. Cellular stress in various forms, particularly oxidative, can suspend nuclear transport and lead to cell death. Prolonged oxidative stress manifests in myriad conditions, including cancer, viral infection and metabolic disease. An IMP protein, Importin-13 (IMP13), retains function under stress, while all other IMP family members tested to date do not. Phylogenetic and structural analysis revealed Transportin-3 (TNPO3) as the closest homologue of IMP13, suggesting it may also retain its function under stress. Subcellular localisation studies indicated that TNPO3 maintained its typical subcellular localisation, even in the presence of stress, unlike most IMP family members. Also, fluorescence recovery after photobleaching (FRAP) demonstrated that TNPO3 shuttling is unaffected under stress. Co-immunoprecipitation studies examining cargo binding revealed the capacity of TNPO3 to bind its cargo in the presence of stress. This demonstrated for the first time that TNPO3 retains functionality under stress conditions, in contrast to other IMPs, but similar to IMP13. Furthermore, both IMP13 and TNPO3 appear to protect against the potentially critical mislocalisation of Ran, a key molecule involved in the maintenance of the nuclear transport system.

## 1. Introduction

Eukaryotic cell survival is dependent on the separation of the nucleus and cytoplasm which regulates access to the cell’s genetic information. The segregation of nuclear and cytosolic compartments protects critical biomolecules such as DNA. Processes such as transcription necessitate controlled access to the DNA held within the nucleus, therefore requiring a tightly regulated protein transport system [1,2]. The complexities of nuclear transport are well characterised, with the proteins that mediate nuclear transport being part of a superfamily known broadly as Importins (IMPs) [3,4,5]. Importin-β (IMPβ), of which there are multiple homologues, is the primary mediator of transport, while Importin-α (IMPα) homologues act as adaptor proteins that can augment the recognition of cargo by IMPβ1, but are not essential for transport [6]. Depending on the direction of transport, importins are found either in the nucleus or cytoplasm, typically in the perinuclear region. Nuclear import occurs when an IMPβ protein, referred to as an importin, binds to an import cargo through interaction with a specialised sequence known as a nuclear localisation sequence, or NLS [7]. The importin can then bind to and translocate through a multiprotein complex embedded within the nuclear envelope, known as the nuclear pore complex (NPC). Once inside, dissociation of the protein–cargo complex is stimulated by the small GTPase protein Ran in its GTP-bound state, binding to the importin [3,8]. The importin is then recycled into the cytoplasm to repeat the process. Nuclear export is an analogous process, with the main differences being that the IMPβ protein, often referred to as an exportin, interacts with cargo via a nuclear export sequence (NES) in a trimeric complex with RanGTP within the nucleus before exiting via the NPC. Once in the cytoplasm, RanGTP is hydrolysed to RanGDP, leading to dissociation of the Exportin:Ran:cargo complex. The Ran cycle is therefore an essential element of nuclear transport, as it provides directionality for IMPβ movement within the cell [9]. Ras-related nuclear protein, or Ran, is a small GTPase protein that exists in two forms: an active GTP-bound form or an inactive GDP-bound form. The overall nuclear transport mechanism is reliant on a gradient, wherein a high concentration of RanGTP is found in the nucleus, with a subsequently low concentration in the cytoplasm, and vice versa for RanGDP. Nuclear transport is thought to be almost completely reliant on this gradient for movement into and out of the nucleus, as it mediates both binding to and dissociation of IMPβ proteins from their cargo [10].

When a cell is exposed to certain stress conditions, a cellular fate decision is activated that, if strong enough, will induce cell death via apoptosis or necrosis [11]. Pathways such as caspase activation are prioritised over nuclear transport if the insult tips the balance toward cell death. As a result, the Ran gradient is disrupted and Ran exits the nucleus, disturbing the cycle that maintains typical nuclear transport [12]. Many different forms of stress have been shown to induce these conditions [13,14,15]. The onset of oxidative stress, typically through exposure to exogenous reactive oxygen species (ROS), causes the breakdown of many cellular structures and processes, as cells often do not have the capacity to counter excess ROS [16]. Endogenous ROS, such as free radicals, play an essential role in the host defence system and various signalling cascades. However, if cells have excess ROS, it can lead to severe consequences, including DNA damage, protein misfolding and membrane damage [17]. Much work has been done to investigate the changes in nuclear trafficking in response to oxidative stress and delineate which processes are rendered non-functional [18,19,20,21]. These conditions can impact the accessibility and ability of NPC residues (known as Nups) to interact with IMPβ proteins, broadly affecting nuclear transport. Under severe oxidative stress conditions, IMPβ1/α mediated nuclear transport is drastically impacted; however, specific cell death pathways are induced that require access to the nucleus by transcription factors to initiate either cell death or repair, suggesting nuclear transport is still able to occur through an alternate pathway [18,22]. This seemingly paradoxical phenomenon is potentially explained by a unique member of the IMPβ family that retains its ability to transport under stress: Importin-13 (IMP13).

Despite the abrogation of classical nuclear transport under stress, IMP13 not only maintains its function, but plays a major role in the oxidative stress response [23]. Gajewska et al. demonstrated that IMP13 binds to and transports two key molecules, SP1 and KLF4, which are involved in the transcriptional response to oxidative stress that promotes cell death. Under these conditions, IMP13 does not mislocalise, unlike other IMPβ family members. Interestingly, the study shows that when IMP13 and/or SP1/KLF4 are absent from cells, the cell is resistant to the effects of oxidative stress. IMP13 has been shown to continue to transport other cargo under stress, particularly proteins like nCLU [23]. nCLU is a pro-apoptotic protein involved in cell death, which interacts with KU70, a protein involved in the DNA repair pathway [23,24]. The role of IMP13 in regulating the transport of these two proteins implicates IMP13 as a key regulator of cell fate decisions.

Here, we identify the structural and evolutionary similarities between TNPO3 and IMP13 that suggest they may function in a similar way. We tested the hypothesis that TNPO3 functions under oxidative stress by performing multiple assays. Subcellular localisation studies using confocal microscopy investigated the effect of sublethal oxidative stress on TNPO3 localisation, which was found to be unaffected. Following this, we performed fluorescence return after photobleaching and co-immunoprecipitation assays, which support our hypothesis that TNPO3 is functional and can transport cargo under oxidative stress, like IMP13. We also observed that TNPO3 and IMP13 can impact the subcellular localisation of Ran, therefore altering the state of the Ran gradient and potentially having a protective effect.

## 2. Materials and Methods

### 2.1. Cell Culture and Transfection

HeLa cells cultured in EMEM base media (Sigma Aldrich, St. Louis, MO, USA) supplemented with 1% non-essential amino acids, 2 mM GlutaMAX and 10% *v*/*v* heat inactivated foetal bovine serum were maintained at 37 °C in 5% CO_2_ and passaged every 3–4 days. Cells were seeded into 6-well plates containing round glass coverslips (24 mm), 12-well plates containing square glass coverslips (15 × 15 mm) or in 6 cm FluoroDishes^TM^ (WPI, Sarasota, FL, USA) 1–2 days prior to experiments as required. Cell concentrations (cells/mL) were determined using the EVE^TM^ automated cell counter (NanoEntek, Seoul, South Korea). Lipofectamine-3000 (ThermoFisher Scientific, Waltham, MA, USA) was used to transfect plasmids into mammalian cells according to manufacturer’s instructions. The plasmids GFP-IMPβ1, GFP-IMPα2, GFP-IMP7 and GFP-IMP13 were used for mammalian cell expression of GFP-IMPβ1, GFP-IMPα2, GFP-IMP7 and GFP-IMP13, as described previously [18,19]. GFP-TNPO3 and mCh-TNPO3 were purchased from GenScript.

### 2.2. Immunofluorescence Assays

For immunofluorescence experiments, cells plated onto glass coverslips in 12-well plates were fixed in 4% paraformaldehyde and permeabilised with 0.2% Triton X-100, then blocked with 1% BSA in phosphate-buffered saline (PBS). After blocking, cells were stained with primary antibody for 1 h at RT, then stained with secondary antibody for 1 h at RT. Cells were washed with PBS, with 4′,6-diamidino-2-phenylindole (DAPI) (1:10,000) included in the final wash with ddH_2_O, then mounted onto slides using FluoroGel (ProSciTech, Kirwan, QLD, Australia) mounting medium. Antibodies used for immunostaining were primary mouse monoclonal anti-Ran (Cat no. 610340, BD Biosciences, Franklin Lakes, NJ, USA) and secondary Alexa-Fluor 568-goat anti-mouse (Invitrogen, Waltham, MA, USA).

### 2.3. Confocal Laser Scanning Microscopy (CLSM) and Image Analysis

For oxidative stress treatment where indicated, cells were incubated with 125 µM H_2_O_2_ made up in complete EMEM for 1 h at 37 °C in 5% CO_2_. For live experiments, cells were imaged in phenol-free DMEM 18–24 h post-transfection using a 100× oil immersion lens on the Olympus FV1000 confocal laser scanning microscope (Monash Micro Imaging Facility, Rome, Italy). For fixed experiments, slides were imaged using either the Olympus FV1000 confocal laser scanning microscope with a 100× oil lens, the Nikon C1 Invert confocal laser scanning microscope with a 60× or 100× oil lens, or the Leica SP8 Invert confocal laser scanning microscope with a 63× oil lens (Monash Micro Imaging Facility). Digitised microscopic images were analysed using ImageJ 1.53k (National Institutes of Health, Bethesda, MD, USA) to determine the ratio of nuclear (Fn) to cytoplasmic (Fc) fluorescence, where Fn/c = (Fn − Fb)/(Fc − Fb). Fb = background due to autofluorescence.

### 2.4. Fluorescence Recovery After Photobleaching (FRAP)

HeLa cells transfected to express GFP-tagged constructs or co-transfected to express mCh- and GFP-tagged constructs were imaged live using the Olympus FV1000 confocal laser scanning microscope (Monash Micro Imaging Facility) with a 63× oil immersion objective at 37 °C. Cells were treated for stress as indicated in Section 2.2, and FRAP was performed as previously described [23,25]. Briefly, regions of interest (nuclei) were exposed to a high-powered laser for 12 scans at 12.5 ms/pixel, imaged, then observed every 20 s for 500 s for fluorescence recovery. ImageJ was used to analyse the fluorescence of the nucleus and cytoplasm before and after photobleaching to determine the return of nuclear fluorescence resulting from movement/import into the nucleus of fluorescently tagged proteins. Data is represented as fractional recovery (Frec) of fluorescence of the nucleus (Fn) minus the background (Fb), using the following equation: Frec(Fn − b) = (Fn − Fb)/prebleach fluorescence. The initial rate (e.g., fluorescence return over the first 100 s post-bleaching) was determined as a change in Frec(Fn − b).

### 2.5. Co-Immunoprecipitation and Western Blot Analysis

HeLa cells expressing GFP or GFP-SRSF1 were stressed as in Section 2.3. Samples were then treated with Glutaraldehyde to induce protein crosslinking as previously described, then lysed [23,26]. The GFP-Trap^®^ Agarose bead kit from ChromoTek (Proteintech, Rosemont, IL, USA) was used to elute immunocomplexes as per manufacturer’s directions, with input and bound samples analysed using Western blotting. Mouse anti-GFP (1:1000) (Cat no. 11814460001, Roche, Basel, Switzerland) and mouse anti-TNPO3 (1:1000) (Cat no. ab54353, Abcam Limited, Cambridge, UK), and secondary goat anti-mouse IgG HRP (AP308P) and goat anti-rabbit IgG HRP (AP307P) (Invitrogen, Waltham, MA, USA) were used.

### 2.6. Statistical Analysis

Statistical analysis was performed using Student’s *t*-test unless otherwise stated with GraphPad Prism 10.1 software.

## 3. Results

### 3.1. IMP13 and TNPO3 Are Structurally and Evolutionarily Similar

**Figure 1:**
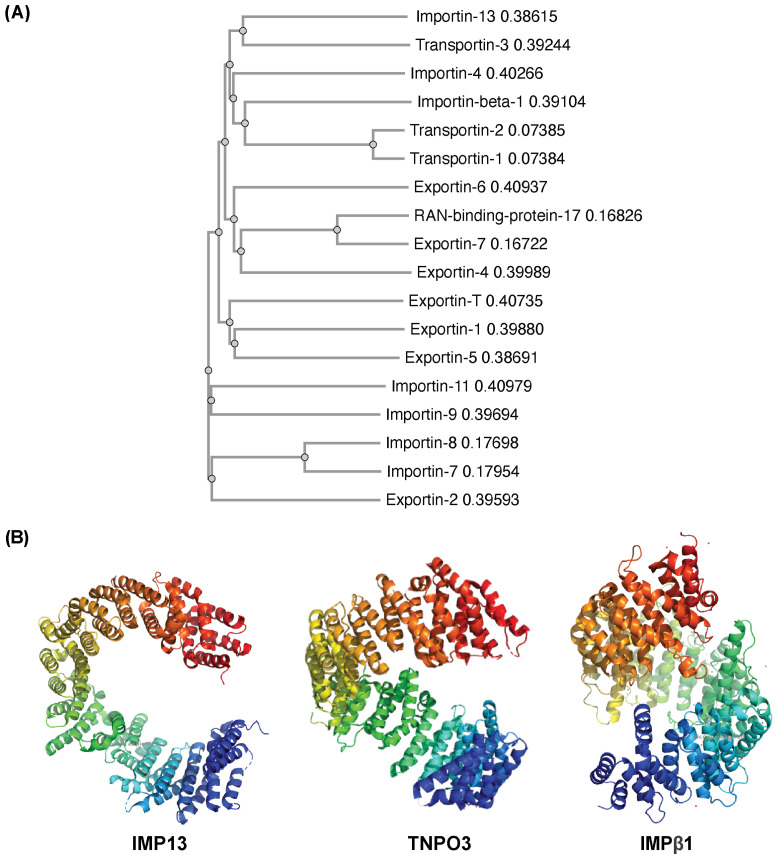
IMP13 and TNPO3 are structurally and evolutionarily similar. (**A**) Phylogenetic tree of human IMPβ family members: Sequences for the indicated IMPβ family members were obtained from National Centre for Biotechnology Information (NCBI), then aligned using Clustal Omega 1.2.4. ClustalX was used to generate the phylogenetic tree. Tree is not drawn to scale; numbers represent distance scores between each sequence. (**B**) Structures of IMP13, TNPO3 and IMPβ1 presented as a ribbon representation using Pymol, coloured from red at the C-terminus to blue at the N-terminus. PDB files; IMPβ1: 1QGK, IMP13: 3ZKV, TNPO3: 4C0P.

Given the important role IMP13 plays in response to cellular stress, we set out to investigate if other closely related family members had similar roles/properties. A phylogenetic tree constructed of the IMPβ family members (Figure 1A), shows IMP13 clustered considerably far away from the rest of the family, along with only one other protein: Transportin-3 (TNPO3). Previous studies had determined the two proteins to be paralogous, meaning that they likely share the same ancestor and have evolved to bind and transport different cargo over time, with over 20% amino acid sequence identity between the two proteins [27,28]. An evolutionary trace analysis conducted by Kimura et al., 2021 determined that, while there are many similarities in sequence between IMP13 and TNPO3, they differ considerably in specific positions, particularly cargo-binding regions [28]. A study by Quan et al. found that TNPO3 and IMP13 may originate from a common ancestor protein, MTR10, in *S. cerevisiae*, suggesting that though they may have evolved to transport different cargoes, they may function under the same conditions [5].

Structurally, classical members of the IMPβ family such as Importin-β1 (IMPβ1) are composed of ~19 repeating structural units referred to as HEAT repeats, which are characterised by a hairpin of two α-helices connected by a short loop [29,30,31]. The compact yet mobile structure (Figure 1B), described as ‘snail-shaped’, is characterised by its ability to change conformation dramatically upon binding cargo and/or RanGTP, as it essentially engulfs its binding partner for transport [32,33]. This conformation is made rigid upon binding with RanGTP, which stabilises the protein–cargo complex [33]. Unlike the prototypic IMPβ1 that contains 19 HEAT repeats, IMP13 is composed of 20 repeats, and adopts a more open, circular conformation than that of IMPβ1 (Figure 1B). In terms of binding to its cargo, IMP13 does not recognise traditional NLSs or NESs; rather, it is thought to bind instead through conformational complementarity [34]. This unique feature enables it to bind many different cargoes through its ability to adapt to each cargo at every stage of transport [28,35]. The distinctive structure and mechanism of binding compared to traditional members of the IMPβ family is presumably what allows it to perform transport bidirectionally for such a wide range of cargo. TNPO3, like IMP13, contains 20 HEAT repeats, and exists in an open, toroidal form (Figure 1B) [27]. One hypothesis considered for TNPO3 was that, rather than binding cargo through an NLS, it bound through RS domains (regions on the cargo that are highly phosphorylated), as it transports primarily SR proteins, named after their long stretches of repeats of serine and arginine residues [27]. However, recent studies have shown that many cargoes that bind TNPO3 do not contain RS-rich segments nor an NLS, but instead bind via an RNA recognition motif (RRM) found on their cargo, or through shape complementarity, similar to IMP13 [28,36]. Overall, TNPO3 is more structurally similar to IMP13 than other classical IMPβ family members like IMPβ1. Despite this, the cargo transported by TNPO3 and IMP13 differs notably. TNPO3 is primarily an import protein for splicing factors, with some evidence supporting its ability to function as a mediator of export, suggesting it may also be a bidirectional transporter like IMP13 [27,37]. ASF1/SF2, its most well-known and well-characterised cargo, is a primary regulator of alternative splicing that ensures accuracy of splicing by preventing exon skipping at the end of transcription [38,39,40,41]. Given the significant role of IMP13 in stress-responsive nuclear transport, as well as its broad functions in cell survival and maintenance, the closely related TNPO3 was deemed an important candidate to investigate for a potential complementary role in the context of stress-responsive nuclear transport.

### 3.2. Like IMP13, TNPO3 Maintains Typical Localisation Under Oxidative Stress

**Figure 2:**
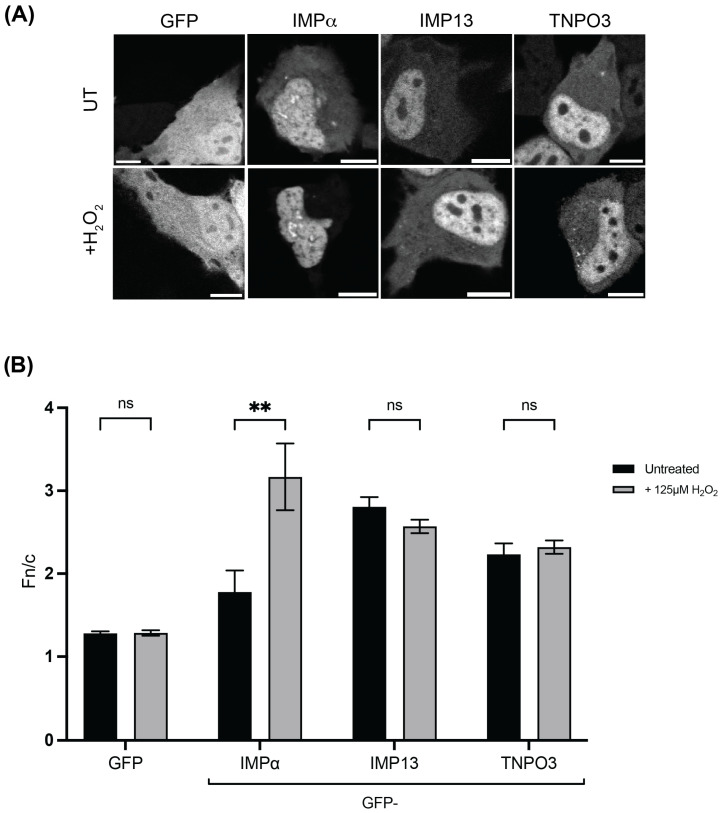
TNPO3 maintains its typical localisation despite the presence of oxidative stress. (**A**) Representative images of HeLa cells transfected to express the indicated GFP-tagged constructs, treated without (UT) or with (+) 125 µM H_2_O_2_ for 1 h prior to live cell imaging using CLSM. Scale bar = 10 µm. (**B**) Quantitative analysis of images such as those shown in A to determine the Fn/c ratio was performed using ImageJ software version 1.53k as per Section 2. Data represents the mean ± SEM (n > 29 cells per sample) from a single typical experiment from a series of three similar experiments. Student’s *t*-test used to determine significance; ns, *p* > 0.05; **, *p* < 0.01.

One of the key unique features of IMP13 is its ability to continue to effectively traffic cargo in the presence of stress, including oxidative stress, while other IMP family members are mislocalised and rendered non-functional [18,21]. To determine if TNPO3 also possesses this ability, we assessed the subcellular localisation of TNPO3 in the presence and absence of oxidative stress by confocal laser scanning microscopy by CLSM. In this study, the stress chosen for investigation is oxidative, and all future experimental references to ‘stress’ refer to oxidative stress induced by H_2_O_2_. Essentially all members of the IMP superfamily of proteins are markedly mislocalised in response to stress, with IMP13 the only identified exception, as it maintains normal localisation [23]. HeLa cells transfected to express either GFP alone or GFP-tagged IMPα, IMP13 or TNPO3 were exposed to 125 mM H_2_O_2_ for 1 h, and imaged live using CLSM. As expected, cells expressing GFP alone exhibited a diffuse localisation between the cytoplasm and the nucleus regardless of the stress state, consistent with GFP having no active transport signals and instead localising by passive diffusion. On the other hand, GFP-IMPα, typical of most IMP family members, demonstrated a striking change in localisation upon exposure to stress. Under basal conditions, GFP-IMPα localised to both the cytoplasm and the nucleus, with a slightly stronger localisation in the nucleus observed. Under oxidative stress, a strong mislocalisation completely to the nucleus was observed (Figure 2A), consistent with previous reports and indicative of non-functional IMPα, which would normally be required to be in the cytoplasm to bind new import cargo [42]. In contrast, GFP-IMP13 and GFP-TNPO3 were both predominantly nuclear in the absence of stress and remained so in the presence of oxidative stress, with no evidence of mislocalisation. This is consistent with previous observations for IMP13 but represents a novel finding for TNPO3 and suggests that it, like IMP13, maintains its subcellular distribution during stress, and may therefore also retain its transport functionality [23]. These qualitative results were confirmed quantitatively by analysis of the digitised microscopic images to determine the nuclear-to-cytoplasmic fluorescence ratio (Fn/c) for each sample (Figure 2B) (see Section 2). An Fn/c > 1 indicates nuclear accumulation, whereas an Fn/c < 1 indicates nuclear exclusion. As expected, GFP did not demonstrate a significant change in localisation in response to stress (Fn/c = 1.3 vs. 1.3; *p* = 0.9275), whereas GFP-IMPα exhibited a significant increase in nuclear accumulation under stress conditions (Fn/c of 1.8 in the absence of stress, compared to a value of 3.2, n = 29, in its presence, *p* < 0.005). In contrast, GFP-IMP13 (Fn/c values of 2.8 and 2.6 in the absence and presence of stress, respectively, *p* = 0.1074) and GFP-TNPO3 (Fn/c values of 2.2 and 2.3 respectively, *p* = 0.5751), did not demonstrate a significant change in response to stress (Figure 2B), consistent with the qualitative observations (Figure 2A).

### 3.3. TNPO3 Maintains Shuttling Capacity Under Oxidative Stress

**Figure 3:**
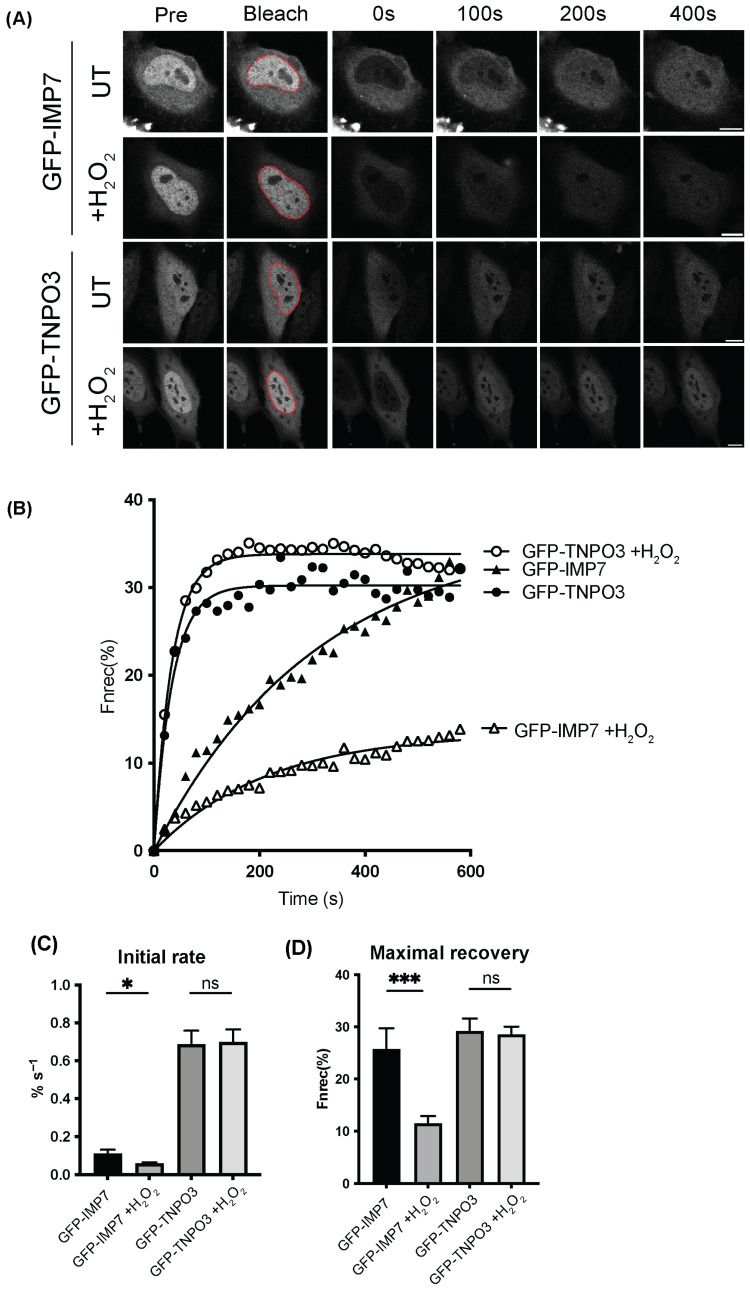
TNPO3 continues to shuttle between the cytoplasm and the nucleus under oxidative stress. (**A**) HeLa cells transfected to express GFP-TNPO3 or GFP-IMP7 were imaged by CLSM prior (Pre) to photobleaching of the cell nucleus (indicated by the dashed red lines in (**A**)), then monitored for recovery every 20 s for 500 s. (**B**) Digitised images, such as those shown in (**A**), were analysed to determine the initial rate of import (initial 100 s post-bleaching). (**C**) The maximal recovery of nuclear fluorescence (**D**). Bar charts represent the mean ± SEM (n > 13 for each condition). Statistical analysis used Student’s *t*-test. ns *p* > 0.05 * *p* < 0.05, *** *p* < 0.001. Scale bars, 10 μm.

Next, the kinetics of TNPO3 transport under stress were examined using FRAP, as our previous study published in Cells demonstrated IMP13 retaining the ability to traffic to the nucleus under stress using this technique [23]. HeLa cells were transfected to express either GFP-TNPO3 or GFP-IMP7, an IMPβ family member that has decreased transport efficiency and is mislocalised under stress, and exposed to 125 µM H_2_O_2_ for 1 h. The nuclei of transfected cells were then bleached using a high-powered laser (conditions outlined in Section 2) and monitored for 600 s. Representative images of pre-bleached cells and the same cells 0, 100, 200 and 400 s after treatment are shown in Figure 3A, where GFP-TNPO3 appears to recover swiftly under stress in comparison to GFP-IMP7-expressing cells, which do recover, but to a lesser extent. Quantitative analysis of digitised versions of each cell over the time course was performed. Interestingly, the speed of GFP-TNPO3 shuttling into the nucleus was unaffected by oxidative stress, while there was a significant decrease in the speed of GFP-IMP7 as determined by the initial rate in the first 100 s (Figure 3B,C). Similarly, there was no change in the maximal recovery of GFP-TNPO3 regardless of stress conditions, whereas IMP7 demonstrates a drastic reduction in recovery to the nucleus (Figure 3D). GFP-TNPO3 can therefore be said to maintain its ability to shuttle under stress, in comparison to GFP-IMP7, which is significantly impaired.

### 3.4. TNPO3 Binds Cargo SRSF1 in a Cellular Context Under Oxidative Stress

**Figure 4:**
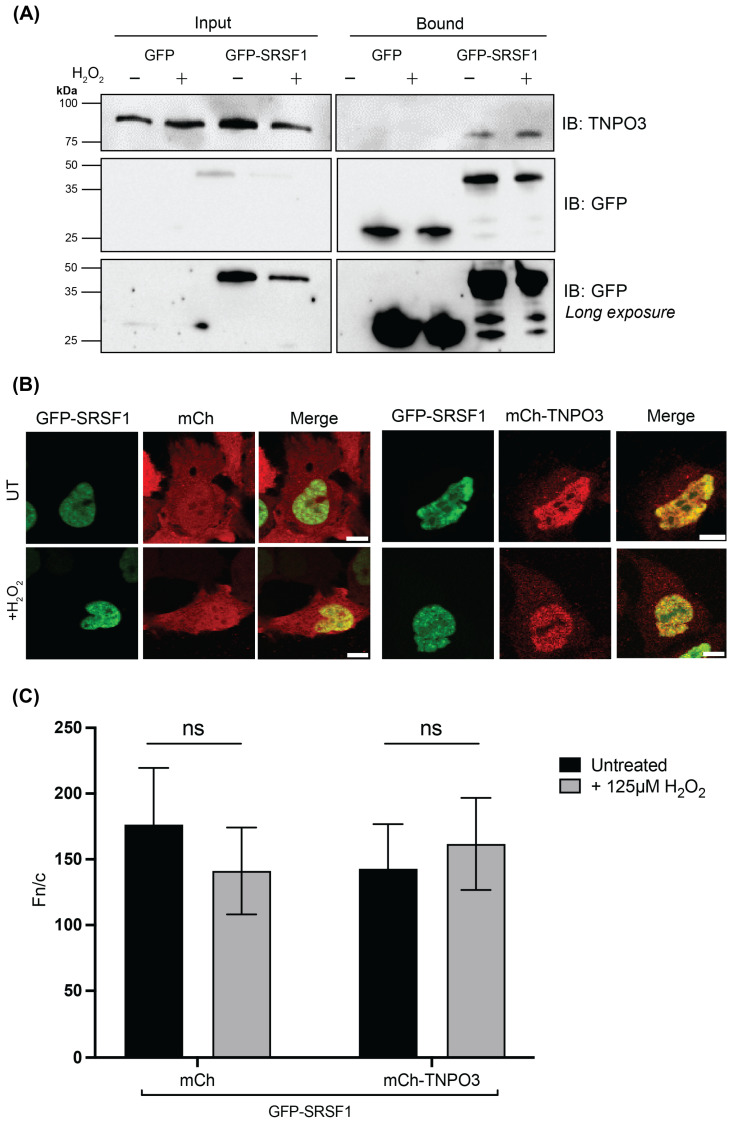
TNPO3 maintains binding with its cargo SRSF1 in a cellular context under oxidative stress. (**A**) HeLa cells transfected to express either GFP or GFP-SRSF1 were treated without or with 125 µM H_2_O_2_, then crosslinked for 3 h using 0.01% glutaraldehyde. Cells were lysed and co-immunoprecipitation using GFP-Trap beads was performed. Samples were separated using SDS-PAGE and Western blotting performed using anti-TNPO3 and anti-GFP antibodies as indicated. Molecular weight markers are indicated. (**B**) Representative images of HeLa cells co-transfected to express GFP-SRSF1 (coloured green) and either mCherry (mCh) or mCh-TNPO3 (coloured red), treated without or with 125 µM H_2_O_2_, prior to imaging live using CLSM. Scale bar = 10 µm. (**C**) Quantitative analysis of images such as those shown in (**A**) to determine the Fn/c ratio was performed using ImageJ software as per Section 2.6. Data represents the mean ± SEM (n > 30 cells per sample) from a single typical experiment from a series of 3 similar experiments. Student’s *t*-test used to determine significance; ns, *p* > 0.05.

As we have demonstrated that TNPO3 maintains localisation and shuttling capacity in the presence of stress, the next step was to investigate its capacity to mediate transport under these conditions. SRSF1 (or ASF/SF2) is a primary cargo of TNPO3, and as it has been studied extensively, but not under oxidative stress, it was chosen for this study. HeLa cells transfected to express either GFP alone or GFP-SRSF1 were treated without or with 125 µM H_2_O_2_ for 1 h, then crosslinked using 0.01% glutaraldehyde to enhance the stability of transient interactions between proteins, as any RanGTP in the sample can cause the IMP/import cargo complex to dissociate during cell lysis. The cells were lysed, and co-immunoprecipitation was performed using the GFP-Trap^®^ system, according to the manufacturer’s instructions. The input and bound samples were separated by SDS-PAGE and analysed by Western blot to probe for co-immunoprecipitation of TNPO3 using anti-TNPO3 and anti-GFP primary antibodies (Figure 4A).

Endogenous TNPO3 was pulled down by GFP-SRSF1 in both the absence and presence of stress, while no TNPO3 was present in the GFP alone bound samples. This indicates that TNPO3 and SRSF1 are in complex with one another in a cellular context in either the presence or absence of stress. This contrasts what would typically be observed in an IMPβ:cargo interaction, which would be significantly diminished or lost altogether under stress [23]. This data supports our hypothesis that TNPO3 likely maintains functionality as a transporter under stress, in a similar fashion to the closely related IMP13.

Studies by Baade et al. and Gajewska et al. showed IMP13 overexpression greatly impacted the localisation of many different cargo proteins, suggesting that the concentration of IMP13 is the rate-limiting factor in many of its transport interactions. This was examined for TNPO3 using steady-state co-transfection experiments with TNPO3 and SRSF1 (Figure 4B,C) [23,43]. HeLa cells co-transfected to express GFP-SRSF1 and either mCh or mCh-TNPO3 were treated without or with 125 µM H_2_O_2_ for 1 h, then imaged using CLSM. As expected, mCh alone expression did not significantly impact the localisation of GFP-SRSF1, which remained extremely nuclear (Fn/c of 176), as is typical of its wildtype localisation due to its role as a splicing factor, with no significant change under oxidative stress (Fn/c of 141). Interestingly, mCh-TNPO3 also had no effect on GFP-SRSF1 localisation, suggesting that transporter availability is not the rate-limiting factor of this known cargo/carrier interaction and that endogenous levels of TNPO3 are sufficient for correct SRSF1 localisation. Similarly, under stress conditions, no changes in GFP-SRSF1 in these samples were observed (Fn/c of 142 and 161, without and with stress, respectively).

**Figure 5:**
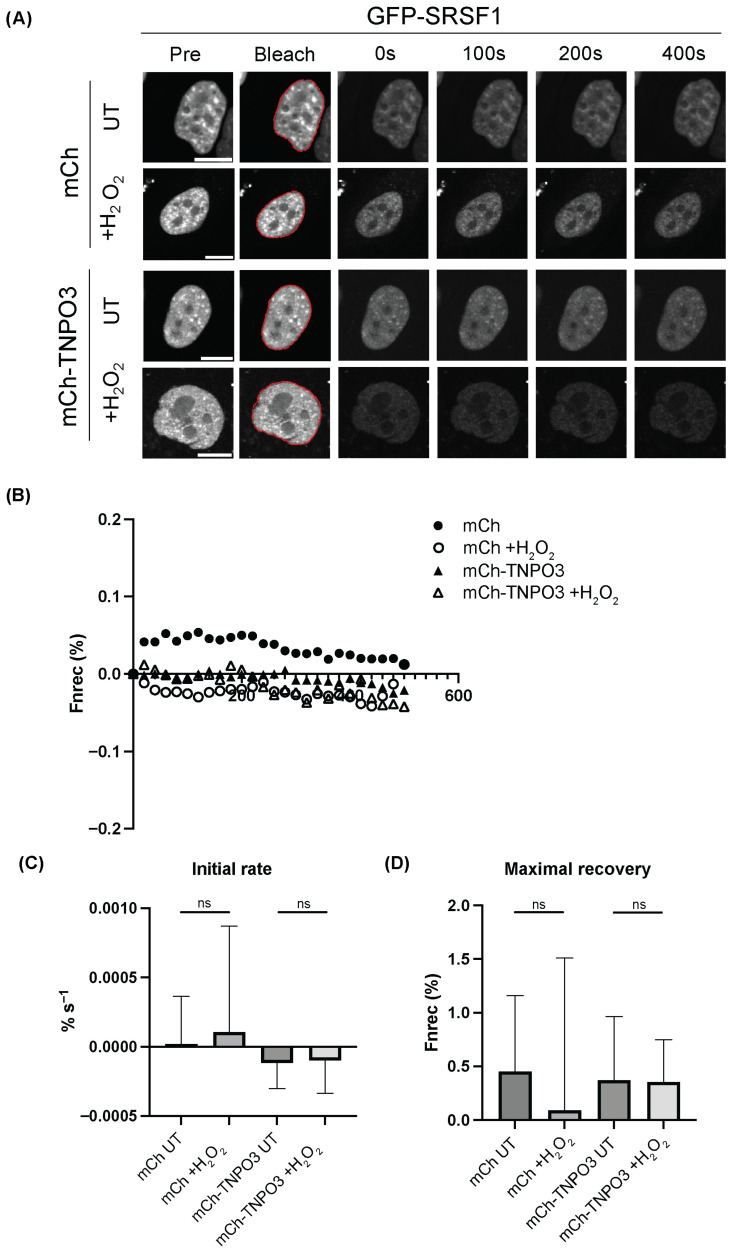
Overexpression of TNPO3 with/without oxidative stress does not impact SRSF1 nuclear import. (**A**) CLSM images of HeLa cells transfected to co-express either mCherry or mCh-TNPO3 with GFP-SRSF1 and treated without or with 125 µM H_2_O_2_ for 1 h. Cells were imaged prior (Pre) to photobleaching of the cell nucleus (indicated by the dashed red lines in (**A**)), then monitored for recovery every 20 s for 500 s, (**B**) Digitised images, such as those shown in (**A**), were analysed to determine the initial rate of import (initial 100 s post-bleaching, (**C**)), the maximal recovery of nuclear fluorescence. (**D**) Bar charts represent the mean ± SEM (n > 14 for each condition). Statistical analysis used Student’s *t*-test. ns, *p* > 0.05 Scale bars, 10 μm.

In addition, we assessed the kinetics of SRSF1 shuttling by TNPO3 under stress, again using FRAP. HeLa cells co-transfected to express GFP-SRSF1 and either mCh or mCh-TNPO3 were treated without or with 125 µM H_2_O_2_ for 1 h, with the nuclei of transfected cells then being bleached using a high-powered laser (conditions outlined in Section 2) and monitored for 600 s. Representative images of pre-bleached cells and the same cells at 0, 100, 200 and 400 s after treatment are shown in Figure 5A. There was no significant difference in the speed or rate of recovery of GFP-SRSF1 under either condition or with mCh or mCh-TNPO3 overexpression, suggesting that there is perhaps no change in SRSF1 shuttling under stress, nor when TNPO3 is in excess (Figure 5C,D).

### 3.5. TNPO3 and IMP13 Modulate the Level of Ran Localising in the Nucleus and Its Recovery Post-Oxidative Stress

**Figure 6:**
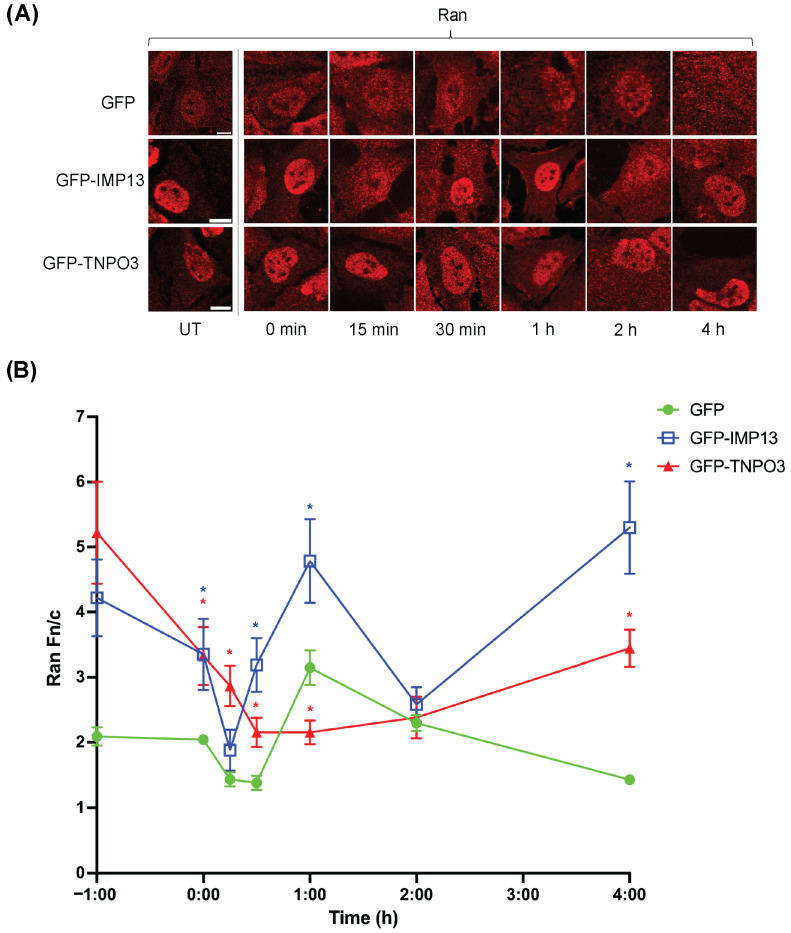
TNPO3 and IMP13 modulate the level of Ran localising in the nucleus and its recovery post-oxidative stress. (**A**) HeLa cells transfected to express GFP, GFP-IMP13 or GFP-TNPO3 and imaged before (UT) or after treatment with 125 µM H_2_O_2_. Samples were fixed at the indicated time intervals, stained for Ran (coloured red) and imaged using CLSM. Scale bar = 10 µm. See Figure S2 for DAPI and GFP channels. (**B**) Quantitative analysis of Fn/c of Ran in GFP transfected cells as shown in (**A**). Data represent the mean ± SEM (n > 22 per sample) from a single typical experiment from a series of 3 similar experiments. Student’s *t*-test determined significance, * *p* < 0.05 compared to GFP alone sample at the same timepoint.

As the maintenance of the Ran gradient is an important indicator of cellular stress, we next investigated the effect of GFP-TNPO3 and GFP-IMP13 expression on the Ran gradient. This was achieved by monitoring the extent of Ran nuclear localisation, since Ran mislocalisation to the cytoplasm is indicative of the collapse of the Ran gradient [12]. HeLa cells transfected with GFP, GFP-TNPO3, or GFP-IMP13 were treated without or with 125 µM H_2_O_2_ for 1 h, then allowed to recover in fresh media for intervals up to 4 h. Representative images of immunostained Ran from cells expressing GFP or GFP-tagged constructs in Figure 6A were analysed to determine the level of nuclear localisation of Ran using the Fn/c parameter (Figure 6B). GFP-expressing cells demonstrated a relatively typical display of Ran mislocalising in the cytoplasm upon exposure to stress, with a Ran Fn/c of ~1.4 at 15 and 30 min. After this, the cells seem to recover/overcompensate for stress, with Ran localising strongly in the nucleus (e.g., Fn/c > 3 at 1 h post-stress), but drop back down to an Fn/c ~1.4 at the 4 h time point. By comparison, GFP-IMP13- and GFP-TNPO3-expressing cells retained significantly higher amounts of Ran in the nucleus, (Fn/c values of 4.2 ± 0.59 and 5.2 ± 0.78 for GFP-IMP13 and -TNPO3, respectively), seemingly due to ectopic expression of the higher amounts of the two proteins.

Strikingly, although the overall trend in terms of fluctuations in Ran nuclear localisation in GFP-IMP13-expressing cells in response to stress and during recovery was broadly similar to that of GFP-expressing cells, Ran remained strongly nuclear (Fn/c > 2) throughout (lowest Fn/c of ~2 at 15 min post-stress, recovering to > 3 at 30 min post stress), implying that although Ran nuclear localisation was impacted, it never reached a critically low level, representative of a collapsed Ran gradient. By 4 h, the level of nuclear accumulation had returned to the high level (Fn/c > 4) corresponding to its starting point. This implies that ectopic expression of IMP13 may provide a buffer to the Ran gradient, protecting against collapse in response to sublethal levels of oxidative stress.

GFP-TNPO3-expressing cells revealed a distinct but comparable scenario where Ran mislocalisation to the cytoplasm appeared to be reduced throughout, although in this case it is also delayed, with the lowest level of Ran nuclear localisation not attained until 1 h post-stress (Fn/c of 2.2), compared to 15 min in the case of GFP-alone-expressing cells (see above). GFP-TNPO3-expressing cells also did not exhibit the apparent rebound spike after ~1 h that was observed in the other lines. Similar to GFP-IMP13-expressing cells, the lowest level of Ran nuclear localisation did not approach critically low levels at any timepoint (Fn/c > 2.2 throughout). Therefore, as observed for IMP13, ectopic expression of TNPO3 appears overall to provide a buffer to the Ran gradient, protecting against collapse. Overall, the fact that Ran in GFP-IMP13- and -TNPO3-expressing cells only minimally reached an Fn/c ~2 indicates that conventional nuclear transport is likely to remain at least partially functional in these stressed cells. Both IMP13 and TNPO3 therefore appear to protect against critical mislocalisation of Ran; TNPO3 also appears to delay the Ran mislocalisation response and IMP13 appears to accelerate its recovery. Together, these results suggest that both TNPO3 and IMP13 play a protective role for the Ran gradient in response to stress.

## 4. Discussion

### 4.1. TNPO3 Retains the Ability to Transport Cargo, Even Under Stress

This study first investigated the structural and evolutionary similarities between IMP13 and TNPO3 that suggested they may also function in similar ways (Figure 1). In humans, IMP13 regulates an important stress-induced cell death/repair pathway, when typical IMPβ proteins are rendered nonfunctional, suggesting the intriguing possibility that TNPO3 also functions under oxidative stress conditions. Cells are continuously subjected to oxidative stress, and defective responses to this burden are integral to pathologies associated with cancer, metabolic disease, viral infection, and muscular dystrophy [44,45,46,47]. TNPO3 has been implicated in viral infection, particularly in HIV-1 infection through the nuclear import of the HIV-1 integrase protein [36,37,48,49]. HIV-1 infection has been shown to promote ROS production and induce oxidative stress within infected patients [50]. In the case where TNPO3 remains active under these conditions, it is thus a useful target to investigate in the prevention and treatment of HIV-1 infection. Interestingly, a mutation of TNPO3 that results in a rare form of muscular dystrophy, LGMDD2, has been experimentally shown to confer resistance to HIV-1 infection, demonstrating the importance of understanding how this protein functions [51]. We believe an investigation of cellular responses to oxidative stress is the crucial first step in broadening our understanding of such pathologies and the mechanisms involved.

It was determined through steady-state and kinetic analysis that TNPO3 localisation and transport capacity remain unchanged in the presence of stress (Figure 2 and Figure 3). Our FRAP data suggests that the rate of TNPO3 nuclear import and its capacity to fully recover to the nucleus are also unaffected by these conditions, in contrast to that observed for other IMP:cargo complexes (Figure 3) [23]. As TNPO3 maintains functional capacity under stress, it is likely that it may also play an important role in the broader oxidative stress response, particularly as its primary role is in transporting splicing factors to the nucleus. Alternative splicing (AS), performed by the SR family of proteins, is a fundamental process in all species required for the response to stress [52,53]. Interestingly, in *A. thaliana*, the TNPO3 homologue MOS14 is thought to be essential for immune regulation through its role in the import of such proteins, suggesting an evolutionary role for TNPO3 in cellular responses to stress [54]. Logically, the next step was to determine the capacity of TNPO3 to bind to its cargo under stress conditions. SRSF1 was chosen for this study as it is a well-studied import cargo of TNPO3, and a key SR protein that performs AS [55]. We demonstrated that TNPO3 remained bound to SRSF1, even under stress, and the cargo localisation remained unchanged (Figure 4). As there is no difference in the localisation of GFP-SRSF1 when cells undergo oxidative stress, this suggests that TNPO3 can continue importing SRSF1 under such conditions. Utilising FRAP, it was found that the shuttling of SRSF1 is unaffected (Figure 5). Due to the volume discrepancy between the nucleus and the cytoplasm, where the nucleus is less than 10% of the cell’s volume, even very low levels of cytoplasm protein (below the visible detection limit) are able to recover once concentrated in the nucleus. The fact that no recovery is shown demonstrates that we are not seeing a change in transport of SRSF1. While it is possible that there is no cytoplasmic pool of protein to be transported to the nucleus, the main conclusion we observed is that SRSF1 is not suddenly exported in response to stress, and we did not see a dramatic response to stress in the form of protein recovery. This is consistent with our argument that oxidative stress is not affecting the transport of this protein. Surprisingly, overexpressing TNPO3 did not impact the localisation or kinetics of SRSF1 nuclear transport, suggesting that TNPO3 is not the rate-limiting factor in the process of SRSF1 nuclear import. It would prove fruitful to investigate the effect of TNPO3 knock down/out on SRSF1 localisation and kinetics, however no studies to date have successfully knocked out TNPO3 in human cells while retaining cellular viability. In *C. elegans*, even knocking down the homologue of TNPO3 is embryonic lethal, suggesting that the protein is essential for viability [56]. Combined, these results suggest that TNPO3 trafficking of SRSF1 is maintained under oxidative stress conditions when other import pathways are rendered non-functional.

### 4.2. TNPO3 and IMP13 Overexpression Modulates the Ran Gradient, Preventing Collapse

Ran is a key driver of nucleocytoplasmic transport, with its mislocalisation from nucleus to cytoplasm indicative of abrogation of conventional IMP-dependent transport. As IMP13 is less reliant on RanGTP for binding and releasing cargo, it follows that when the gradient collapses, IMP13 can still function [23]. Since we have demonstrated that TNPO3 retains shuttling and cargo-binding capacity under these same conditions, it therefore may also be less dependent on RanGTP binding for cargo transport. While the precise underlying mechanism remain as yet unknown, TNPO3 likely interacts with cargo in both a Ran-dependent and -independent fashion, enabling it to perform various roles under both normal and stress conditions. The specific cargo and interactions critical to this newly discovered function in Ran gradient regulation should be the subject of further study. In addition, given that the other IMPs are rendered non-functional by stress conditions, it is possible that IMPs such as TNPO3 and IMP13, which retain efficient transport capacity, may be involved in actively mediating Ran mislocalisation or recovery. As both proteins are functional without Ran, we believed it would be interesting to investigate whether they have any impacts on the gradient itself, which has never previously been investigated for an IMPβ protein under oxidative stress. Strikingly, we discovered that overexpressing either GFP-IMP13 or GFP-TNPO3 resulted in a significant mislocalisation of Ran into the nucleus under normal cellular conditions (Figure 6), suggesting that both transporters contribute to increasing its nuclear accumulation, potentially buffering the cell from stress-induced collapse of the Ran gradient. Although no abnormal cell death was observed when these proteins were overexpressed, whether elevated Ran nuclear localisation is itself detrimental under basal conditions does warrant further investigation. Importantly, TNPO3 and IMP13 may buffer or protect against critical mislocalisation of Ran under oxidative stress conditions. At almost every time point post stress treatment, cells overexpressing GFP-TNPO3 or GFP-IMP13 maintained a high level of nuclear Ran, suggesting mitigation of the effect of oxidative stress that results in Ran mislocalisation. Previous studies have shown that stress impacts intracellular ATP availability, which in turn alters the ability of the Ran guanine nucleotide exchange factor RCC1 to convert GDP to GTP, resulting in a lower concentration of RanGTP in stressed cells [12]. Whether overexpressing TNPO3 or IMP13 may modulate the relative RanGTP/RanGDP levels is unclear, as the only commercial antibodies currently available for immunofluorescent staining target total Ran rather than individual bound isotypes. A RanGTP-specific antibody has been reported, but it is not suitable for immunofluorescence. It would also be interesting to assess whether the protective effect is enough to allow other members of the IMPβ superfamily, such as IMPβ1, to continue to mediate nuclear transport under stress when TNPO3 or IMP13 are present at high levels. From this, either or both proteins may in fact contribute to the establishment of the Ran gradient, as the IMP13 cargo protein Ubc9 has previously been shown to be connected to this process when expressed in the nucleus [57]. As we believe many TNPO3 cargoes remain undiscovered, it may indeed potentially transport cargoes that have similar roles in the formation and maintenance of the Ran gradient. These findings are extremely novel, considering how little is known about regulation of the Ran gradient. Future research could include investigating the collapse of the Ran gradient under other forms of stress (e.g., heat or osmotic), and how homologues of IMP13 and TNPO3 from other species contribute to its maintenance.

The differential yet intertwined roles of IMP13 and TNPO3 provide a unique perspective to investigate cellular responses to oxidative stress, as nucleocytoplasmic transport is the backbone that facilitates entry into the nucleus to induce downstream cellular effects. IMP13 has an established role in the oxidative stress transcriptional response, as it mediates the nuclear export of two key transcription factors, specificity protein 1 (SP1) and Kruppel-like factor 4 (KLF4) [58]. Intriguingly, knocking down IMP13 in combination with either SP1 or KLF4 contributed to resistance of embryonic stem cells against stress-induced death, suggesting that IMP13 tips the balance from survival toward cell death. If TNPO3 is the counterbalance to IMP13, therefore promoting survival, this may explain why it is so difficult to generate knockout cell lines of both proteins. Furthermore, IMP13 has been shown to regulate apoptosis through the clusterin (CLU)/KU70 axis. KU70 is exported to the cytoplasm by IMP13 where it binds pro-apoptotic protein Bax, preventing it from activating apoptosis. Under conditions of stress, IMP13 imports nCLU, which binds KU70, preventing it from blocking Bax activation, which initiates apoptosis [23]. Considering the essential role of IMP13 in these processes, it is highly likely that TNPO3 is involved in the regulation of stress responses in a similar way. As TNPO3 is a key regulator of alternative splicing due to its role in the nuclear import of key splicing factors, it may perhaps regulate a different stress pathway to that of IMP13, providing an avenue for future research. Elucidating the role of TNPO3 in the broader context of cellular stress is key for understanding how nuclear transport can be modulated to tip the delicate balance of cellular fate in whichever direction benefits the organism, by either promoting the survival of healthy cells, or the removal of harmful ones.

## Data Availability

The original contributions presented in this study are included in the article/Supplementary Material. Further inquiries can be directed to the corresponding authors. The raw data supporting the conclusions of this article will be made available by the authors on request.

## Supplementary Materials

The following supporting information can be downloaded at: https://www.mdpi.com/article/10.3390/cells15080708/s1, Figure S1: Whole blots representing TNPO3 co-immunoprecipitation from Figure 4A; Figure S2: Representative images in DAPI, GFP and DsRed2 channels from the time course shown in Figure 6.

## Abbreviations

The following abbreviations are used in this manuscript:

CLSM	Confocal Laser Scanning Microscopy
FRAP	Fluorescence Return After Photobleaching
H_2_O_2_	Hydrogen peroxide
IMP	Importin
IMP13	Importin-13
IMP7	Importin-7
IMPα	Importin-α
IMPβ	Importin-β
NES	Nuclear Export Sequence
NLS	Nuclear Localisation Sequence
NPC	Nuclear Pore Complex
Ran	RAs-related Nuclear Protein
ROS	Reactive Oxygen Species
SRSF1	Serine and Arginine rich Splicing Factor 1
TNPO3	Transportin-3
