# Supplementary figures and images for: The Nuclear Transporter Transportin-3 Functions Under Oxidative Stress
Source: Cells. 2026 Apr 17;15(8):708. doi: 10.3390/cells15080708 (PMC13114304; doi:10.3390/cells15080708)

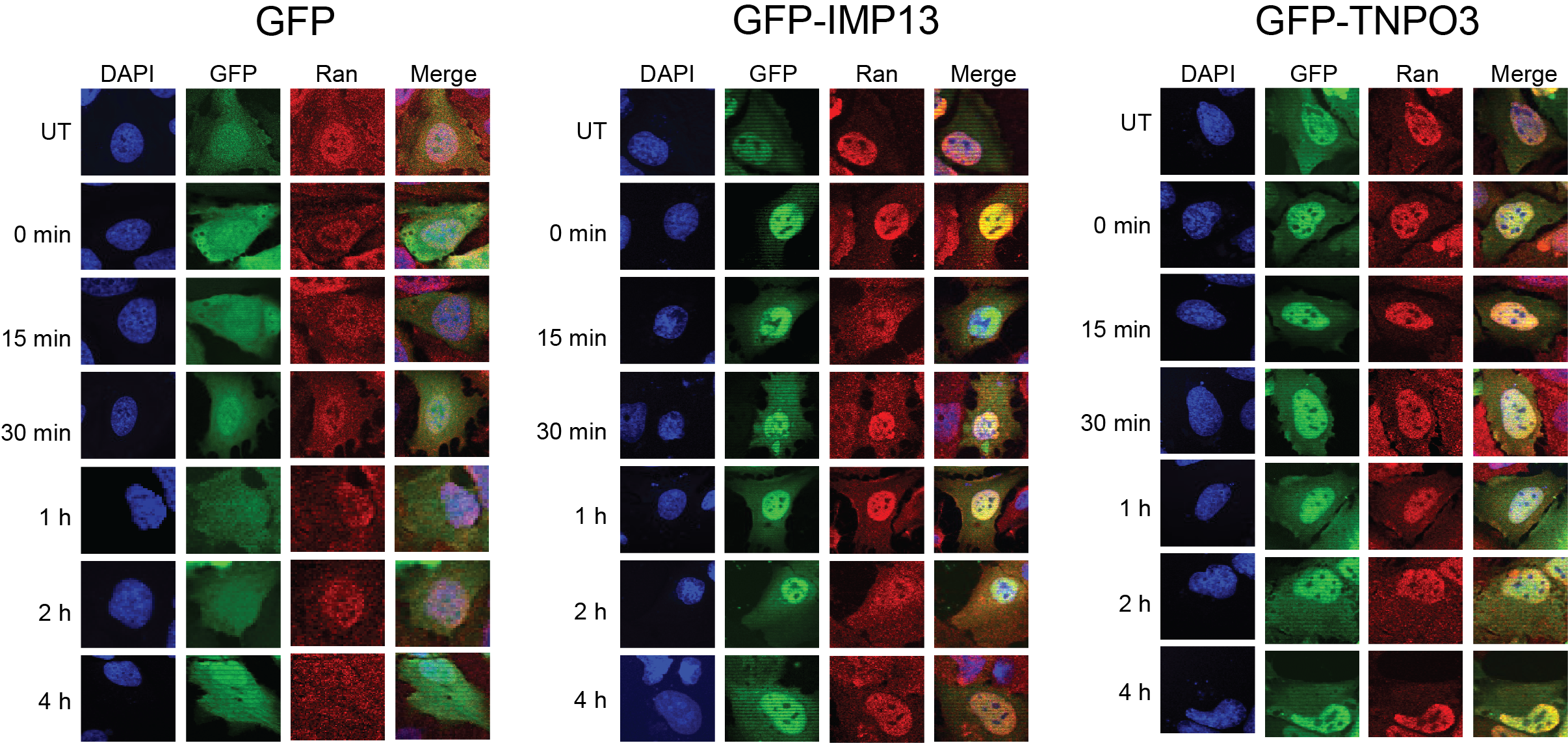

Supplement: Supplementary file 1 [file cells-15-00708-s001.zip › Supplementary Figures/SupplementaryFigure2.png]

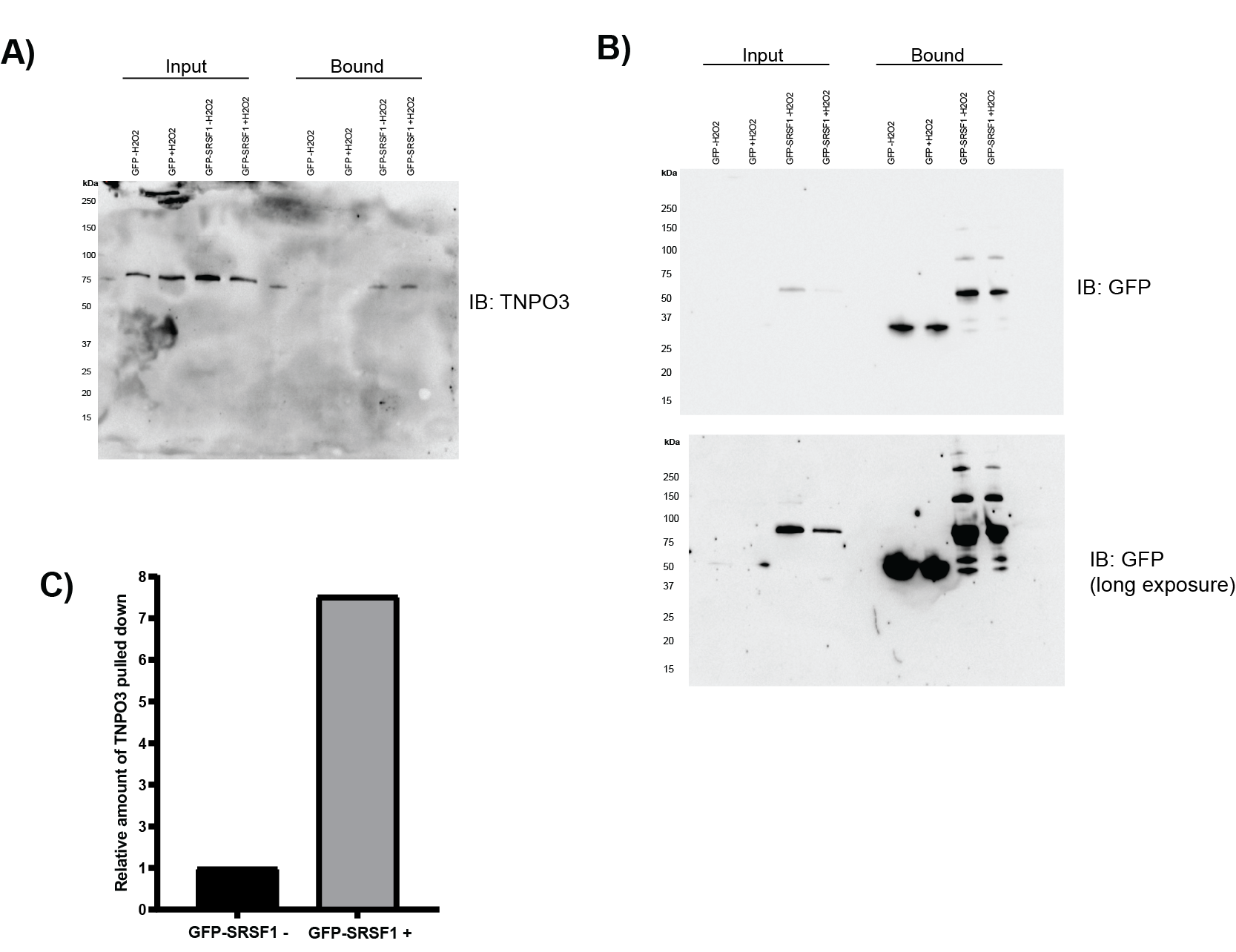

Supplement: Supplementary file 1 [file cells-15-00708-s001.zip › Supplementary Figures/SupplementaryFigure1.png]
